# The Case of General Grant

**Published:** 1885-04

**Authors:** 


### THE CASE OF GENERAL GRANT.

We need not commend to the thoughtful consideration of every dentist the article upon the condition of General Grant, written at our urgent request, and published in this number. It will attract the attention of all. It is not offered as a contribution to the sensational literature of the day, nor to gratify a morbid curiosity. The case is one of great professional interest, and it is full of instruction to both dentist and physician. That the oral condition may be, and often is, a prime factor in inducing malignant tumors of the mouth, is a fact that cannot be too quickly and thoroughly comprehended. General Grant was a smoker, and every anti-tobacco crank in the country immediately jumps to the conclusion that the disease that is slowly but surely eating out the life of the most distinguished man of his age is the direct fruit of the cigar; whereas, aside from the fact that the smoker's mouth is seldom in a cleanly condition, tobacco probably had little or nothing do to with the origin of the tumor. The case should most certainly be thoroughly investigated from the dental standpoint, that it may prove of greatest benefit to others, for this instance is not at all unique. In the next number we shall publish, from a competent source, an article upon epithelioma, which cites more than one analogous case.

